# Proyecto Mamá: a lifestyle intervention in overweight and obese Hispanic women: a randomised controlled trial – study protocol

**DOI:** 10.1186/s12884-015-0575-3

**Published:** 2015-07-30

**Authors:** Lisa Chasan-Taber, Bess H. Marcus, Milagros C. Rosal, Katherine L. Tucker, Sheri J. Hartman, Penelope Pekow, Edward Stanek, Barry Braun, Caren G. Solomon, JoAnn E. Manson, Sarah L. Goff, Glenn Markenson

**Affiliations:** 1 Department of Biostatistics & Epidemiology, School of Public Health & Health Sciences, 405 Arnold House, University of Massachusetts, 715 North Pleasant Street, Amherst, MA 01003-9304 USA; 2 Department of Family Medicine and Public Health, School of Medicine, University of California San Diego, San Diego, USA; 3 Division of Preventive and Behavioral Medicine, Department of Medicine, University of Massachusetts Medical School, Worcester, MA USA; 4 Department of Clinical Laboratory & Nutritional Sciences, University of Massachusetts Lowell, Lowell, MA USA; 5 Department of Kinesiology, School of Public Health & Health Sciences, University of Massachusetts, Amherst, MA USA; 6 Brigham and Women’s Hospital and Harvard Medical School, Boston, MA USA; 7 Department of Medicine and the Center for Quality of Care Research, Baystate Medical Center, Springfield, MA USA; 8 Division of Maternal-Fetal Medicine, Baystate Medical Center, Springfield, MA USA

**Keywords:** Lifestyle intervention, Randomised controlled trial, Healthy eating, Prevention, Diet, Latina, Physical activity, Postpartum, Pregnancy, Gestational diabetes mellitus, Overweight, Obesity

## Abstract

**Background:**

The proportion of women entering pregnancy overweight or obese has been rising and, in turn, is associated with adverse maternal and fetal outcomes. Gestational weight gain (GWG) exceeding Institute of Medicine (IOM) guidelines further increases health risks and has been independently associated with postpartum weight retention. Hispanic women are disproportionately affected by overweight and obesity, but have had limited access to interventions that promote healthy lifestyles due to cultural, socioeconomic, and language barriers. Therefore, the overall goal of this randomized controlled trial is to test the efficacy of a culturally and linguistically modified, individually-tailored lifestyle intervention to reduce excess GWG, increase postpartum weight loss, and improve maternal metabolic status among overweight/obese Hispanic women.

**Methods/Design:**

Overweight/obese Hispanic women are recruited in early pregnancy and randomly assigned to a Lifestyle Intervention (n = 150) or a Comparison Health and Wellness (control) intervention (n = 150). Multimodal contacts (i.e., in-person, telephone counseling, and mailed print-based materials) are used to deliver the intervention from early pregnancy (12 weeks gestation) to 6 months postpartum, with follow-up to 1 year postpartum. Targets of the intervention are to achieve IOM Guidelines for GWG and postpartum weight loss; American Congress of Obstetrician and Gynecologist guidelines for physical activity; and American Diabetes Association guidelines for diet. The intervention draws from Social Cognitive Theory and the Transtheoretical Model and includes strategies to address the specific social, cultural, and economic challenges faced by low-income Hispanic women. Assessments are conducted at baseline (~10 weeks gestation), mid pregnancy (24–28 weeks gestation), late pregnancy (32–34 weeks gestation) and postpartum at 6-weeks, 6-months, and 12-months by bicultural and bilingual personnel blinded to the intervention arm. Efficacy is assessed via GWG, postpartum weight loss, and biomarkers of glycemic control, insulin resistance, and cardiovascular disease risk factors. Changes in physical activity and diet are measured via 7-day accelerometer data and 24-h dietary recalls at each assessment time period.

**Discussion:**

Hispanic women are the fastest growing minority group in the U.S. and are disproportionately affected by overweight and obesity. This randomised trial uses a high-reach, low-cost strategy that can readily be translated into clinical practice in underserved and minority populations.

**Trial registration:**

NCT01868230 May 29, 2013

## Background

Weight gain in women of reproductive age is increasing, contributing to a rise in the proportion of women entering pregnancy overweight or obese [1]. In turn, overweight and obesity in pregnancy are established risk factors for such maternal complications as cesarean delivery, hypertension, gestational diabetes mellitus (GDM), and preeclampsia [2–4]. Gestational weight gain (GWG) exceeding Institute of Medicine (IOM) guidelines further increases health risks and has been independently associated with postpartum weight retention [5]. In addition, recent reports indicate a relationship between high GWG, an abnormal metabolic environment in utero, increased risk of large-for-gestational-age (LGA) infants [6], and subsequent childhood adiposity and morbidity [7].

In the United States, more than 40 % of pregnant women exceed the Institute of Medicine (IOM) guidelines for GWG [1]. Studies have observed high rates of excessive GWG among Hispanic women with, for example, 52 % of overweight and 75 % of obese Hispanic women having excessive GWG [8, 9]. This finding is noteworthy as Hispanics are the largest minority group in the U.S., with the highest birth and immigration rates of any minority group [10]. Hispanics from the Caribbean islands (i.e., Puerto Ricans and Dominicans) are the 2^nd^ largest group of Hispanics living in the U.S.[10], the fastest growing subgroup, and the largest Hispanic subgroup in the Northeast U.S.[11]. As compared to other Hispanics, Puerto Ricans and Dominicans experience the greatest health disparities, the highest prevalence of type 2 diabetes, and exhibit more adverse behaviors such as poor nutrition [12–14]. Half as many Hispanic women comply with recommendations for physical activity during pregnancy as compared to non-Hispanic whites [15]. Hispanics are disproportionately affected by overweight and obesity; at each BMI level, Hispanics have a higher prevalence of diabetes than non-Hispanic whites [16–18]. However, due to cultural factors, socioeconomic circumstances, differences in educational background, and language barriers, Hispanics have had limited access to clinical and public health interventions that promote healthy lifestyles.

The IOM has noted that existing research is inadequate to establish the characteristics of interventions that work reliably to assist women in meeting guidelines for GWG [1] and recent meta-analyses have been unable to identify the defining features of effective interventions [19, 20]. Interventions which begin during pregnancy [21] may be more effective than those initiated only in the postpartum period [22–24] given that it may be difficult to reduce postpartum weight retention without first preventing excessive GWG during pregnancy [25]. There is also evidence that both energy intake restriction and physical activity are needed to reduce weight in the postpartum period [23, 26] compared to exercise alone [27]. Therefore, the overall objective of this trial is to test the ability of a lifestyle intervention, informed by formative behavioral research, to reduce excess GWG, increase postpartum weight loss, improve maternal metabolic status among Hispanic women entering pregnancy overweight or obese Hispanic as well as to improve adiposity measures among their offspring.

### Aims

Specific aims for mothers are to achieve IOM Guidelines for GWG, postpartum weight loss, and improve maternal metabolic status by achieving and maintaining 1) GWG within guidelines based on pre-pregnancy BMI [1]; 2) a 5 % reduction from pre-pregnancy weight, 3) at least 150 min per week of moderate intensity physical activity such as brisk walking, as recommended by the American Congress of Obstetrician and Gynecologists (ACOG) [28], and 4) reduction in total energy intake by following a balanced healthy diet as recommended by American Diabetes Association (ADA) [29]. Specific aims for offspring are to improve adiposity measures (i.e., weight, waist and head circumference, skinfold thicknesses, and fetal growth). The final aim evaluates the cost-effectiveness of the lifestyle intervention (i.e., start-up costs, total cost/participant, and ratio of average dollars spent per average incremental improvement in outcome.

## Methods

**Fig. 1:**
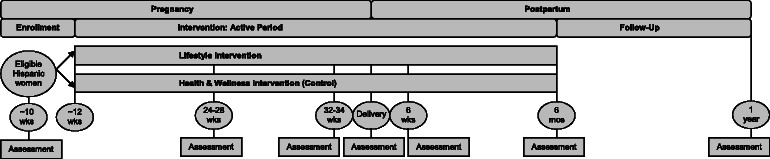
Trial Design

Proyecto Mamá is based at the ambulatory obstetrical practices of Baystate Medical Center in Western Massachusetts. Eligible pregnant Hispanic women are recruited in early pregnancy (~10 weeks gestation) and randomly assigned to a Lifestyle Intervention (n = 150) or a Comparison Health & Wellness (control) Intervention (n = 150) (Fig. 1). Multimodal contacts (i.e., in-person, telephone counseling, and mailed print-based materials) are used to deliver the intervention from ~12 weeks gestation to delivery (Phase 1: Pregnancy Intervention) and from 6 weeks to 6 months postpartum (Phase 2: Postpartum Intervention); follow-up continues to 1 year postpartum (Fig. 1).

### Eligibility criteria

Eligible women are pregnant Hispanic women who are overweight or obese (BMI ≥ 25 kg/m^2^) and 18–45 years of age. Women with the following characteristics are excluded: 1) pre-pregnancy BMI < 25 kg/m^2^, 2) history of type 2 diabetes, heart disease, or chronic renal disease, 2) contraindications to participation in moderate physical activity or to a low-fat/high-fiber diet (e.g., Crohn’s disease, ulcerative colitis), 3) inability to read English or Spanish at a 6^th^ grade level, 4) <16 or >45 years of age, 5) >16 weeks gestation, 6) current medications which adversely influence glucose tolerance, 7) not planning to continue to term or deliver at the study site, or 8) non-singleton pregnancy (e.g., twins, triplets, etc.).

### Recruitment

The trial capitalizes on our experience with culturally appropriate strategies for recruiting pregnant Hispanic women in practice-based settings [30]. Specifically, bilingual (Spanish and English) and bicultural health educators recruit women at the first prenatal care visit (~10 weeks gestation). Women are informed of the aims and procedures of the project and asked to sign a written informed consent form, as approved by the Institutional Review Boards of the University of Massachusetts-Amherst and Baystate Health. Upon enrollment, the baseline assessment includes: 1) data collection of behaviors during pregnancy via standardized questionnaires, 2) measures of physical activity via an ActiGraph GT3X-plus activity monitor (Actigraph LLC, Pensacola, FL) to be worn on the wrist for the next 7 days, 3) measures of dietary intake via three unannounced 24-h dietary recalls over the following two-week period, and 4) laboratory measures of biomarkers of glycemic control, insulin resistance, and other cardiovascular disease risk factors via a study-specific fasting blood sample. After completion of each assessment and blood draw, participants receive a gift card.

### Randomisation

Randomisation to the Lifestyle Intervention or to the Health & Wellness (control) Intervention occurs at ~12 weeks gestation, after completion of the baseline assessment. Randomisation is stratified based on age (<30, ≥30 years) and pre-pregnancy BMI (overweight ≥25- < 30 kg/m^2^ vs. obese ≥30 kg/m^2^). Within each strata, a blocked randomization is used such that both treatment groups are assigned an equal number of times in each set of 4 sequentially enrolled patients.

### Lifestyle intervention

The Lifestyle Intervention is an evidence-based approach utilizing culturally and linguistically modified, motivationally targeted, individually-tailored intervention materials developed in our prior randomised controlled trials among Hispanics [31–38]. The intervention draws from Social Cognitive Theory [39] and the Transtheoretical Model [40] and takes into account findings by our research group on the specific social, cultural, economic, and environmental resources, as well as challenges faced by women of Hispanic backgrounds [41–43]. An initial pilot study, Estudio Vida, conducted among 68 overweight and obese Hispanic women found that the lifestyle intervention was feasible [44].

The Prenatal Phase (~12 weeks gestation - delivery) (Fig. 1) starts with a face-to-face introductory session building upon the usual prenatal care received by patients. The goal of this phase is to optimize GWG; the health educator sets a GWG goal based on pre-pregnancy BMI and explains the importance of meeting weight gain guidelines. The weight-based curriculum focuses on periodic weight monitoring, graphing and education. Participants are provided with a digital scale and are encouraged to weigh themselves at home daily and to chart their weight on a grid weekly. Emphasis is placed on using the scale as an important feedback and learning tool for how to better regulate personal diet and exercise behaviors. Motivational interviewing principles are used to identify and strengthen women’s motivations for change.

The introductory session additionally includes administration of physical activity and dietary questionnaires that facilitate tailoring the intervention. The Exercise Tailoring Questionnaire consists of 3 measures: Stages of Change for Physical Activity [45], Processes of Change for Physical Activity [46], and Self-Efficacy for Physical Activity [45]. Physical activity change is targeted via individualized week-by-week goals that focus on increasing, by 10 %, the time spent in moderate-intensity activity as well as steps taken per day. Women choose which form of safe activity they enjoy the most or can most readily fit into their lifestyle, from dancing to walking in a shopping mall to yard work. The accumulation of short bouts of moderate activity (i.e., 10 min episodes) is encouraged. Pedometers and activity logs are provided for women to monitor their activity. Based on responses to the tailoring questionnaires, individually-tailored computer Expert-System Feedback Reports [36, 38, 47, 48] draw particular messages from a library of approximately 296 messages regarding motivation, self-efficacy, and cognitive and behavioral strategies for exercise adoption. Manuals matched to their stage of motivational readiness focus on the benefits of exercise, building social support for new behavioral patterns, and strategies for overcoming barriers to exercise specific to low-income Hispanic women. Tip Sheets include topics such as stretching and exercising with baby (e.g., walking while pushing a stroller).

The Diet Tailoring Questionnaire is a two-part survey consisting of a checklist of high-calorie and lower-calorie foods and beverages commonly consumed by the target population. Respondents are asked to indicate the frequency that they consume the various foods and to rate their confidence (self-efficacy) in their ability to decrease high calorie and increase low calorie food consumption. Dietary change is targeted by working toward an ultimate goal of 1,500 calories per day (up to 2,000 for breastfeeding women who lose more than 2 lbs/week over a 2 week period). Dietary goals are tailored to the participant based on responses to the Diet Tailoring Questionnaires, which take into consideration energy-dense foods that the participant eats frequently and feels confident that she is able to reduce or replace, and lower calorie foods that the participant eats infrequently and feels confident that she can increase. Participants are instructed in how to self-monitor dietary intake using the food calorie guide and a tip sheet for measuring portion sizes. Participants are provided with measuring cups and a dietary log.

The introductory session is followed over the remainder of pregnancy by biweekly and monthly mailed, print-based materials as well as telephone booster calls to provide motivationally-based individualized feedback. Based on responses to monthly mailed tailoring questionnaires, culturally and motivationally targeted individualized reports are sent. All materials are written at a 6^th^ grade reading level in Spanish or English depending upon participant preference. A third trimester face-to-face session (~34 weeks gestation), with the goal of preparation for the Postpartum Phase, targets knowledge and attitudes regarding postpartum weight loss and type 2 diabetes prevention.

The Postpartum Phase (6 weeks postpartum – 6 months postpartum) (Fig. 1) starts with a face-to-face session. The tailoring questionnaires are repeated and physical activity goals, dietary goals, and a weight loss goal, based on pre-pregnancy BMI, are set. Participants are encouraged to work toward this goal by focusing on a reduction of 1–2 pounds per week. The health educator utilizes a checklist of motivators for weight loss specific to new mothers, to help women identify their own weight loss motivations and to reinforce engagement. Participants are, again, encouraged to weigh themselves at home daily and to chart their weight on a grid weekly.

This postpartum session is followed by monthly mailed, print-based materials as well as telephone booster calls to provide motivationally-based individualized feedback. Booster telephone sessions facilitate: 1) review of progress toward dietary, physical activity and weight loss goals, 2) problem-solving of challenges faced in achieving goals (e.g., balancing caregiver/household responsibilities, cultural norms of self-sacrifice, limited social support, partner negotiation, and neighborhood safety), 3) discussion of mailed tip sheets, and 4) assistance with setting new goals.

Quality control procedures ensure that stage of change and social cognitive constructs are consistently represented in all intervention materials.

### Health & wellness (Control) intervention

To ensure retention and to control for contact time, the Health & Wellness (control) arm receives mailed health materials and telephone booster calls at the same frequency as the Lifestyle Intervention arm. Mailed materials focus on non-exercise and non-dietary topics and include booklets from ACOG and the American Academy of Pediatrics in English or Spanish. These booklets represent high-quality, standard, low-cost, self-help material currently available to the public. In this way, we control for contact time, while keeping the content of the two interventions distinct. Hispanic control-arm participants in our prior studies reported that these materials were of interest and differential dropout did not occur between study arms [35, 49].

### Outcome variables

GWG is calculated as the difference between maternal weight at delivery and pre-pregnancy weight; rate of weight gain is calculated as GWG divided by gestational age at delivery in weeks. Compliance with IOM weight gain guidelines is calculated by comparing the observed weight gain with the 2009 IOM Guidelines [1]. Postpartum weight loss is measured as the difference between weight at 6 or 12 months postpartum and weight at delivery, and calculated as absolute weight change according to pre-pregnancy BMI, percentage who retain a specific amount of weight over pre-pregnancy weight, or proportion whose BMI category changes from pre-pregnancy BMI category (Table 1).

**Table 1:** Variables collected at assessment timepoints

	Pregnancy			Delivery	Postpartum		
	10 weeks	24-28 weeks	32-34 weeks		6 weeks	6 months	1 year
Aim #1: Gestational Weight Gain (GWG) and Postpartum Weight Loss							
GWG (kg)	X	X	X	X			
Rate of GWG	X	X	X	X			
Compliance with IOM guidelines	X	X	X	X			
Area under the GWG curve (AUC)	X	X	X	X			
Postpartum weight loss	X			X	X	X	X
Physical activity (actigraph, PPAQ)	X	X	X		X	X	X
Dietary intake (Hispanic FFQ)	X	X	X		X	X	X
Aim #2: Glycemia and Associated Biomarkers of Insulin Resistance							
Glucose (mg/dl)	X	X	X		X	X	X
Insulin (pmol/l)	X	X	X		X	X	X
Hb A1 c (%)	X	X	X		X	X	X
Adiponectin (μg/ml)	X	X	X		X	X	X
Leptin (ng/ml)	X	X	X		X	X	X
HOMA	X	X	X		X	X	X
Aim #3: Postpartum Biomarkers of Cardiovascular Disease Risk							
Total cholesterol (mg/dL)		X			X	X	X
Triglycerides (mg/dL)		X			X	X	X
HDL-cholesterol (mg/dL)		X			X	X	X
LDL-cholesterol (mg/dL)		X			X	X	X
Systolic blood pressure (mm Hg)	X	X	X		X	X	X
Diastolic blood pressure (mm Hg)	X	X	X		X	X	X
hsCRP (mg/dl)		X			X	X	X
Aim #4: Offspring Outcomes							
Birthweight-for-gestational age z-score [fetal growth]				X			
Ponderal Index (g/cm^3^)				X	X	X	X
Weight-for-length z-score (WFL-z)				X	X	X	X
Weight-for-age z-score (WFA-z)				X	X	X	X
Length-for-age z score (LFA-z)				X	X	X	X
Sum of skinfolds, ratio of skinfolds				X	X	X	X
Waist circumference (cm)				X	X	X	X
Head circumference (cm)				X	X	X	X
Aim #5: Cost Effectiveness Variables							
Star-up costs, total cost/participant	X	X	X	X	X	X	X
Avg $/avg incremental improvement	X	X	X	X	X	X	X
Covariates							
Clinical characteristics of current pregnancy	X	X	X				
Medical history	X						
Sociodemographic factors	X						
Smoking (cotinine) and substance use	X	X	X		X	X	X
Depression	X	X	X		X	X	X
Sleep	X	X	X		X	X	X
Breastfeeding					X	X	X
Social Support	X	X	X		X	X	X

Fasting maternal biomarkers are collected at baseline (~10 weeks gestation), mid pregnancy (24–28 weeks gestation), late pregnancy (32–34 weeks gestation) and postpartum at 6-weeks, 6-months, and 1-year (Table 1). Biomarkers of glycemic control and insulin resistance include glucose, insulin, HbA_1c_, homeostasis model assessment (HOMA), leptin, and adiponectin. Postpartum biomarkers of cardiovascular risk include total cholesterol, LDL-cholesterol, HDL-cholesterol, triglyceride concentrations, systolic and diastolic blood pressure, and C-reactive protein (CRP). Postpartum diabetes screening occurs at each postpartum assessment and follows the guidelines of the 2007 5^th^ International Workshop Conference on GDM recommending a postpartum 75-g OGTT [50] using diagnostic criteria defined by the American Diabetes Association [51].

Child anthropometric measures are obtained within 24 h of delivery, 6 weeks, 6 months, and 1 year postpartum and include measured weight on a calibrated scale, length using a measuring board, waist and head circumference, and subscapular (SS) and triceps (TR) skinfold thicknesses. Fetal growth is calculated as birthweight-for-gestational age z-score and ponderal index (PI) as birth weight (g) x 100/birth length (cm)^3^. Change from 0 to 6 months and from 0 to 12 months in weight-for-length z-score (WFL-z), weight-for-age z-score (WFA-z), and length-for-age z-score (LFA-z) are calculated. Age- and sex-specific scores are calculated using U.S. national reference data (National Center for Health Statistics, CDC Growth Charts).

### Behavioral outcomes

Women are provided with an accelerometer, the ActiGraph GT3X-plus, to be worn on the wrist for 7-days at each of the assessment time periods (baseline, mid pregnancy, late pregnancy, and postpartum at 6-weeks, 6-months, and 12-months). Previous studies have reported reasonable validity under laboratory conditions among pregnant women [52], as well as under free-living conditions [53–55]. In addition, at each assessment time period, trained bilingual personnel blinded to the assigned intervention arm conduct three unannounced 24-h dietary recalls over a two-week period and administer the Pregnancy Physical Activity Questionnaire (PPAQ) [56]. The PPAQ is a semi-quantitative questionnaire, which evaluates participation in household/caregiving, occupational, sports/exercise, and transportation activities.

### Covariates

Clinical characteristics of the current pregnancy and medical history are abstracted from the pregnancy medical record (Table 1). Weight is measured prospectively at each prenatal visit and postpartum weight is measured during assessment visits by trained study staff to the nearest 0.1 kg on accurately calibrated standard clinical scales using a standardized protocol. Gestational age is based upon the best clinical estimate as recorded in the medical record. Gestational age at the time of GDM screen, degree of abnormality on glucose tolerance testing during pregnancy, treatment for glucose abnormality during pregnancy (e.g., diet, oral hypoglycemic agents and/or insulin), pregnancy complications and birth outcomes are abstracted from the medical record.

Sociodemographic factors are collected via self-report at enrollment. Acculturation is assessed via the Psychological Acculturation Scale [57], language preference, and generation in the U.S. Smoking is assessed via the biomarker cotinine. Social support for diet and exercise is measured via the Social Support for Diet and Exercise Behaviors (SSDEB) questionnaire during pregnancy and postpartum [58]. Additional factors collected at each postpartum assessment include: The Pittsburgh Sleep Quality Index (PSQI) [59], the Edinburgh Postpartum Depression Scale [60, 61], and the Infant Feeding Questionnaire [62].

### Data analysis

Primary analyses will evaluate differences in the change from baseline in weight gain measures, glycemic control and other biomarkers of insulin resistance and cardiovascular risk factors between the groups (an intent-to-treat analysis). We will use mixed models with random subject effects, including a common mean at baseline for the treatment groups, a period effect, and an intervention by period interaction [63]. The mixed model analysis will enable inclusion of time varying covariates such as breastfeeding behaviors, depression, and sleep, which may vary between baseline and follow-up for individuals. Equivalence of treatment groups will be assessed by comparing distributions of the potential confounders across groups. In addition, we will investigate established risk factors for weight gain (e.g., parity) and glycemic control as well as degree and treatment for abnormality on glucose tolerance testing during pregnancy (e.g., diet, oral hypoglycemic and/or insulin) as potential confounders or effect modifiers. Mixed model analyses will also be used to account for the repeated measures of exercise and diet and will be used to evaluate adoption and maintenance of behavior change in the Lifestyle Intervention group.

We will evaluate the impact of intervention arm on the offspring outcomes using the methods described above. We will also examine GWG and prenatal glycemic factors as predictors of weight gain in early infancy and childhood. In models focused on offspring weight gain as the outcome, we will additionally adjust for fetal growth (i.e., BW/GA z-score).

### Power and sample size

The study is powered to detect mean differences in change in outcome variables from baseline in the Lifestyle Intervention as compared to the Health and Wellness (control) Intervention that are equivalent to 0.35 to 0.40 standard deviations, or a “small-medium” effect size [64] at 80 % power at a 0.05 level of significance. Based on observed rates in our prior and ongoing trials [44, 65], we conservatively expect 10 % of women to leave the area, deliver elsewhere, or withdraw during pregnancy, and therefore plan to recruit 333 women to achieve our goal of 300 participants (150 in each intervention arm) at the time of delivery. Based on this sample size, we have 80 % power to detect as small as a 2.4 % difference in percent change in HbA_1c_ between treatment arms and 80 % power to detect a 30 % reduction in risk for exceeding IOM guidelines.

For offspring outcomes, we calculate that 4 % will be excluded for very early preterm birth (<34 weeks), miscarriage, or stillbirth resulting in 288 children at delivery. For postpartum outcomes, over the following postpartum year, we conservatively project that an additional 8 % of women will be lost to follow-up due to withdrawal or movement out of the area. Women who become pregnant again will be censored. Therefore, we expect 264 women and children at 1 year postpartum. This provides us with the ability to detect 0.35 to 0.40 standard deviations, or a “small-medium” effect size [64] at 80 % power at a 0.05 level of significance for postpartum weight loss, maternal biomarkers of glycemic control, insulin resistance, and cardiovascular disease risk factors, and offspring adiposity measures at one year postpartum.

## Discussion

This trial is innovative in testing a prenatal and postpartum lifestyle intervention designed to reduce excess GWG, increase postpartum weight loss, and improve maternal metabolic status among overweight/obese Hispanic women. Strengths of the study include a culturally specific exercise and dietary intervention delivered via a combination of modalities including in-person, mail, telephone; individualized diet and physical activity goals; the use of psychological theory to drive the intervention; prior experience with recruitment and follow-up in the study population; multidimensional (subjective and objective) measures of physical activity and diet validated in the study population; measures of adiposity in addition to weight; and multiple and fasting measures of serum biomarkers of insulin resistance. Other advances include: 1) inclusion of a significant proportion of low income women, 2) randomization to a Lifestyle Intervention or a comparison Health & Wellness arm, thereby controlling for contact time; 3) a translatable intervention feasible for primary healthcare providers; and 4) follow-up from pregnancy through the postpartum period allowing the evaluation of both maternal and offspring outcomes.

Women receive closer medical attention during the prenatal and postpartum periods than at other times in their adult lives, and are often highly motivated to improve their health to benefit themselves and their children. This pregnancy and postpartum lifestyle intervention capitalizes upon these teachable moments [66].

The low-income Hispanic population targeted by this study will include Spanish speakers (~25 %), a population who have, overall, been excluded from research studies [67] or are underrepresented in research even when recruitment goals include Hispanics [68] despite the greater health challenges they face. The trial includes novel materials developed specifically for this group, thus increasing its innovation.

Investigation of which types of programs benefit pregnant and postpartum women, and identification of women at particularly high-risk, are needed to increase the effectiveness of any prevention efforts. Changes in lifestyle risk factors (e.g., regular exercise, healthy diet) among overweight and obese Hispanic women may reduce risk of obesity and subsequent type 2 diabetes and CVD for both mother and offspring. The public health impact of such lifestyle modifications is likely to be greatest in ethnic groups, such as Hispanics, with a consistently high prevalence of obesity, diabetes, and the highest prevalence of sedentary behavior. This randomised controlled trial uses a high-reach, low-cost strategy, which can readily be translated into clinical practice in underserved and minority populations.

## References

[1] Committee to Reexamine IOM Pregnancy Weight Guidelines (2009). Weight Gain During Pregnancy: Reexamining the Guidelines.

[2] Cedergren M (2006). Effects of gestational weight gain and body mass index on obstetric outcome in Sweden. Int J Gynaecol Obstet.

[3] Guelinckx I, Devlieger R, Beckers K, Vansant G (2008). Maternal obesity: pregnancy complications, gestational weight gain and nutrition. Obes Rev.

[4] Crane JMG, White J, Murphy P, Burrage L, Hutchens D (2009). The effect of gestational weight gain by body mass index on maternal and neonatal outcomes. J Obstet Gynaecol Can.

[5] Knudsen V, Heitmann B, Halldorsson T, Sorensen TIA, Olsen S: Maternal dietary glycaemic load during pregnancy and gestational weight gain, birth weight and postpartum weight retention: a study within the Danish National Birth Cohort. Br J Nutr. 2013;109(8):1471–8. Epub 2012 Aug 21. 10.1017/S0007114512003443 22906835

[6] Siega-Riz A, Viswanathan M, Moos M, Deierlein A, Mumford S, Knaack J (2009). A systematic review of outcomes of maternal weight gain according to the Institute of Medicine recommendations: birthweight, fetal growth, and postpartum weight retention. Obstet Gynecol.

[7] Oken E, Taveras EM, Kleinman KP, Rich-Edwards JW, Gillman MW (2007). Gestational weight gain and child adiposity at age 3 years. Am J Obstet Gynecol.

[8] Chasan Taber L, Silveira M, Lynch K, Pekow P, Solomon C, Markenson G (2014). Physical activity and gestational weight gain in Hispanic women. Obesity.

[9] Siega-Riz AM, Adair LS, Hobel CJ (1994). Institute of Medicine maternal weight gain recommendations and pregnancy outcome in a predominantly Hispanic population. Obstet Gynecol.

[10] U.S. Department of Commerce Economics and Statistics Administration. US Census Bureau (2007). The American Community—Hispanics: 2004.

[11] Centers for Disease Control and Prevention. Age-adjusted percentage of civilian, noninstitutionalized population with diagnosed diabetes, Hispanics, United States, 1980–2007. 7/29/09.

[12] Hajat A, Lucas JB, Kington R (2000). Health outcomes among Hispanic subgroups: data from the National Health Interview Survey, 1992–95. Adv Data.

[13] Himmelgreen DA, Perez-Escamilla R, Peng Y, Angela B (2005). Birthplace, length of time in the U.S., and language are associated with diet among inner-city Puerto Rican women. Ecol Food Nutr.

[14] Melnik T, Hosler A, Sekhobo J, Duffy T, Tierney E, Engelgau M (2004). Diabetes prevalence among Puerto Rican adults in New York City, NY, 2000. Am J Public Health.

[15] Evenson KR, Savitz DA, Huston SL (2004). Leisure-time physical activity among pregnant women in the US. Paediatr Perinat Epidemiol.

[16] Zambrana RE, Logie LA (2000). Latino child health: need for inclusion in the US national discourse. Am J Public Health.

[17] Centers for Disease Control and Prevention (CDC) (2003). Prevalence of physical activity, including lifestyle activities among adults–United States, 2000–2001. MMWR Morb Mortal Wkly Rep.

[18] Anonymous (2004). Health disparities experienced by Hispanics--United States. Morb Mortal Weekly Rep.

[19] Streuling I, Beyerlein A, Rosenfeld E, Hofmann H, Schulz T, von Kries R (2011). Physical activity and gestational weight gain: a meta-analysis of intervention trials. BJOG.

[20] Gardner B, Wardle J, Poston L, Croker H (2011). Changing diet and physical activity to reduce gestational weight gain: a meta-analysis. Obes Rev.

[21] Ferrara A, Hedderson M, Albright C, Ehrlich S, Quesenberry C, Peng T, Feng J, Ching J, Crites Y: A Pregnancy and Postpartum Lifestyle Intervention in Women With Gestational Diabetes Mellitus Reduces Diabetes Risk Factors: A feasibility randomized control trial. Diabetes Care. 2011;34(7):1519–25. doi:10.2337/dc10-2221. Epub 2011 May 3. PMID: 21540430. 10.2337/dc10-2221 PMC3120183 21540430

[22] Leermakers EA, Anglin K, Wing RR (1998). Reducing postpartum weight retention through a correspondence intervention. Int J Obes.

[23] O'Toole ML, Sawicki MA, Artal R (2003). Structured diet and physical activity prevent postpartum weight retention. J Womens Health (Larchmt).

[24] Ostbye T, Krause K, Lovelady C, Morey M, Bastian L, Peterson B (2009). Active Mothers Postpartum: a randomized controlled weight-loss intervention trial. Am J Prev Med.

[25] Kuhlmann AKS, Dietz P, Galavotti C, England L (2008). Weight-management interventions for pregnant or postpartum women. Am J Prev Med.

[26] Lovelady CA, Garner KE, Moreno KL, Williams JP (2000). The effect of weight loss in overweight, lactating women on the growth of their infants. N Engl J Med.

[27] Lovelady CA, Nommsen Rivers LA, McCrory MA, Dewey KG (1995). Effects of exercise on plasma lipids and metabolism of lactating women. Med Sci Sports Exerc.

[28] ACOG Committee Obstetric Practice (2002). ACOG Committee opinion. Number 267, January 2002: Exercise during pregnancy and the postpartum period. Obstet Gynecol.

[29] Anonymous (2002). American Diabetes Association position statement: evidence-based nutrition principles and recommendations for the treatment and prevention of diabetes and related complications. J Am Diet Assoc.

[30] Chasan-Taber L, Fortner RT, Hastings V, Markenson G (2009). Strategies for recruiting Hispanic women into a prospective cohort study of modifiable risk factors for gestational diabetes mellitus. BMC Pregnancy Childbirth.

[31] Rosal M, Ockene I, Restrepo A, White M, Borg A, Olendzki B (2011). Randomized Trial of a Literacy-Sensitive, Culturally Tailored Diabetes Self-Management Intervention for Low-Income Latinos: Latinos en Control. Diabetes Care.

[32] Rosal MC, Lemon SC, Nguyen OH, Driscoll NE, Ditaranto L (2011). Translation of the diabetes prevention program lifestyle intervention for promoting postpartum weight loss among low-income women. Transl Behav Med.

[33] Ockene IS, Tellez TL, Rosal MC, Reed GW, Mordes J, Merriam PA (2012). Outcomes of a Latino community-based intervention for the prevention of diabetes: the Lawrence Latino Diabetes Prevention Project. Am J Public Health.

[34] Rosal MC, Olendzki B, Reed GW, Gumieniak O, Scavron J, Ockene I (2005). Diabetes self-management among low-income Spanish-speaking patients: a pilot study. Ann Behav Med.

[35] Chasan-Taber L, Marcus B, Stanek E, Ciccolo J, Marquez D, Solomon C (2009). A randomized controlled trial of prenatal physical activity to prevent gestational diabetes: design and methods. J Women's Health.

[36] Marcus BH, Lewis BA, Williams DM, Dunsiger S, Jakicic JM, Whiteley JA (2007). A comparison of Internet and print-based physical activity interventions. Arch Intern Med.

[37] Marcus BH, Lewis BA, Williams DM, Whiteley JA, Albrecht AE, Jakicic JM (2007). Step into Motion: a randomized trial examining the relative efficacy of Internet vs. print-based physical activity interventions. Contemp Clin Trials.

[38] Marcus BH, Napolitano MA, King AC, Lewis BA, Whiteley JA, Albrecht A (2007). Telephone versus print delivery of an individualized motivationally tailored physical activity intervention: Project STRIDE. Health Psychol.

[39] Bandura A (1997). Self-Efficacy: The Exercise of Control.

[40] Prochaska JO, DiClemente CC (1983). Stages and processes of self-change of smoking: toward an integrative model of change. J Consult Clin Psychol.

[41] Rosal MC, Borg A, Bodenlos JS, Tellez T, Ockene IS (2011). Awareness of diabetes risk factors and diabetes prevention strategies among a sample of low-income Latinos with no known diagnosis of diabetes. Diabetes Educ.

[42] Neighbors C, Marquez D, Marcus B (2008). Leisure-time physical activity disparities among Hispanic subgroups in the United States. Am J Public Health.

[43] Marquez D, Bustamante E, Bock B, Markenson G, Tovar A, Chasan-Taber L (2009). Perspectives of Latina and non-Latina white women on barriers and facilitators to exercise in pregnancy. Women Health.

[44] Hawkins M, Hosker M, Marcus BH, Rosal MC, Braun B, Stanek EJ, Markenson G, Chasan Taber L: A pregnancy lifestyle intervention to prevent gestational diabetes risk factors in overweight Hispanic women: a feasibility randomized controlled trial. Diabet Med. 2015;32(1):108–15. doi:10.1111/dme.12601. Epub 2014 Oct 29. PMID: 25306925. 10.1111/dme.12601 25306925

[45] Marcus BH, Selby VC, Niaura RS, Rossi JS (1992). Self-efficacy and the stages of exercise behavior change. Res Q Exerc Sport.

[46] Marcus BH, Rossi JS, Selby VC, Niaura RS, Abrams DB (1992). The stages and processes of exercise adoption and maintenance in a worksite sample. Health Psychol.

[47] Marcus BH, Bock BC, Pinto BM, Forsyth LH, Roberts MB, Traficante RM (1998). Efficacy of an individualized, motivationally-tailored physical activity intervention. Ann Behav Med.

[48] Marcus BH, Emmons KM, Simkin-Silverman LR, Linnan LA, Taylor ER, Bock BC (1998). Evaluation of motivationally tailored vs. standard self-help physical activity interventions at the workplace. Am J Health Promot.

[49] Marcus B, Lewis B, King T, Albrecht A, Hogan J, Bock B (2003). Rationale, design, and baseline data for Commit to Quit II: an evaluation of the efficacy of moderate-intensity physical activity as an aid to smoking cessation in women. Prev Med.

[50] Metzger BE (2007). Summary and recommendations of the Fifth International Workshop-Conference on Gestational Diabetes Mellitus. Diabetes Care.

[51] American Diabetes Association (2004). Gestational diabetes mellitus. Diabetes Care.

[52] van Hees V, Renström F, Wright A, Gradmark A, Catt M, Chen K (2011). Estimation of daily energy expenditure in pregnant and non-pregnant women using a wrist-worn tri-axial accelerometer. PLoS ONE.

[53] St-Onge M, Mignault D, Allison D, Rabasa-Lhoret R (2007). Evaluation of a portable device to measure daily energy expenditure in free-living adults. Am J Clin Nutr.

[54] Kozey-Keadle S, Libertine A, Lyden K, Staudenmayer J, Freedson P: Validation of Wearable Monitors for Assessing Sedentary Behavior. Med Sci Sports Exerc. 2011;43(8):1561–7. 10.1249/MSS.0b013e31820ce174 21233777

[55] Ridgers N, Salmon J, Ridley K, O'Connell E, Arundell L, Timperio A (2012). Agreement between activPAL and ActiGraph for assessing children's sedentary time. Int J Behav Nutr Phys Ac.

[56] Chasan-Taber L, Schmidt MD, Roberts DE, Hosmer D, Markenson G, Freedson PS (2004). Development and validation of a Pregnancy Physical Activity Questionnaire. Med Sci Sports Exerc.

[57] Tropp LR, Erkut S, Coll CG, Alarcon O, Vazquez Garcia HA (1999). Psychological Acculturation: Development of a New Measure for Puerto Ricans on the U.S. Mainland. Educ Psychol Meas.

[58] Sallis JF, Grossman RM, Pinski RB, Patterson TL, Nader PR (1987). The development of scales to measure social support for diet and exercise behaviors. Prev Med.

[59] Buysse DJ, Reynolds CF, Monk TH, Berman SR, Kupfer DJ (1989). The Pittsburgh Sleep Quality Index: a new instrument for psychiatric practice and research. Psychiatry Res.

[60] Cox JL, Holden JM, Sagovsky R (1987). Detection of postnatal depression. Development of the 10-item Edinburgh Postnatal Depression Scale. Br J Psychiatry.

[61] Jadresic E, Araya R, Jara C (1995). Validation of the Edinburgh Postnatal Depression Scale (EPDS) in Chilean postpartum women. J Psychosom Obstet Gynaecol.

[62] Lakshman R, Landsbaugh J, Schiff A, Hardeman W, Ong K, Griffin S (2011). Development of a questionnaire to assess maternal attitudes towards infant growth and milk feeding practices. Int J Behav Nutr Phys Ac.

[63] Stanek EJ (1988). Choosing a Pretest-Posttest Analysis. Am Stat.

[64] Cohen J (1988). Statistical Power Analysis for the Behavioral Sciences.

[65] Chasan Taber L, Marcus BH, Rosal MC, Tucker TL, Hartman SJ, Pekow P (2014). Estudio Parto: postpartum diabetes prevention program for hispanic women with abnormal glucose tolerance in pregnancy: a randomised controlled trial - study protocol. BMC Pregnancy Childbirth.

[66] Phelan S (2010). Pregnancy: a "teachable moment" for weight control and obesity prevention. Obstet Gynecol.

[67] Frayne SM, Burns RB, Hardt EJ, Rosen AK, Moskowitz MA (1996). The exclusion of non-English-speaking persons from research. J Gen Intern Med.

[68] Durant R, Davis R, St George DMM, Williams I, Blumenthal C, Corbie-Smith G (2007). Participation in research studies: factors associated with failing to meet minority recruitment goals. Ann Epidemiol.

